# Assessment of genetic diversity in the endangered populations of *Breonadia salicina* (Rubiaceae) growing in The Kingdom of Saudi Arabia using inter-simple sequence repeat markers

**DOI:** 10.1186/s12863-014-0109-4

**Published:** 2014-10-03

**Authors:** Abdel-Rhman Z Gaafar, Fahad Al-Qurainy, Salim Khan

**Affiliations:** Department of Botany and Microbiology, College of Science, King Saud University, Riyadh, 11451 Saudi Arabia

**Keywords:** *Breonadia salicina*, Genetic variation, Genetic structure, Molecular markers, ISSR, Conservation biology

## Abstract

**Background:**

*Breonadia salicina* (Rubiaceae) is a critically endangered plant at the local scale native to southwestern Saudi Arabia. To understand the levels and partitioning of genetic variation across populations and geographical regions of this species, we assessed its genetic diversity using inter-simple sequence repeat (ISSR) markers.

**Results:**

Fourteen ISSR primers selected from 43 primers gave rise to 211 amplified loci, of which 68 were polymorphic. The percentage of polymorphic loci (PPL) at the population level ranged from 17.1 to 23.7%, with an average of 21.3%. Nei’s gene diversity (*h*) and Shannon’s information index (*I*) were 0.086 and 0.125, respectively. At the species level, PPL was 32.2%, while *h* and *I* were 0.116 and 0.172, respectively. A hierarchical analysis of molecular variance revealed a high level of genetic differentiation among populations (17% of total variance, *P* = 0.001), consistent with the gene differentiation coefficient (*G*_ST_ = 0.256). Nevertheless, the evaluated genetic diversity was very low within populations; while relatively high among populations, levels were insufficient for long-term survival. Saudi Arabian accessions were also compared to accessions of a population from Yemen, where the species is more widespread. The Yemeni population also showed low genetic diversity but clustered separately.

**Conclusions:**

*Breonadia salicina* in Saudi Arabia is characterized by low within-population genetic diversity and high among-population genetic differentiation. Based on our findings, this locally endangered species is on the verge of local extinction. The species’ survival depends on successful implementation of suggested strategies for its long-term conservation.

## Background

The conservation of threatened and endangered medicinal species is indispensable for the future of humankind. The flora of Saudi Arabia is rich in diversity, with numerous rare and endangered plant species. The number of threatened species is increasing annually as a result of adverse environmental conditions and anthropogenic activities [1]. Over-exploitation, including habitat destruction, expansion of urban activities, over-grazing, selective species removal, and mutilation, is leading to genetic erosion. Physical syndromes of declining existence, such as fragmented natural habitats, endangerment, thin populations, narrowed genetic diversity, rarity, paucity of regeneration, and reproductive inefficiencies, are prevalent in the kingdom of Saudi Arabia [2] and may lead to the eventual local extinction of *B. salicina* and many other endangered species.

**Figure 1:**
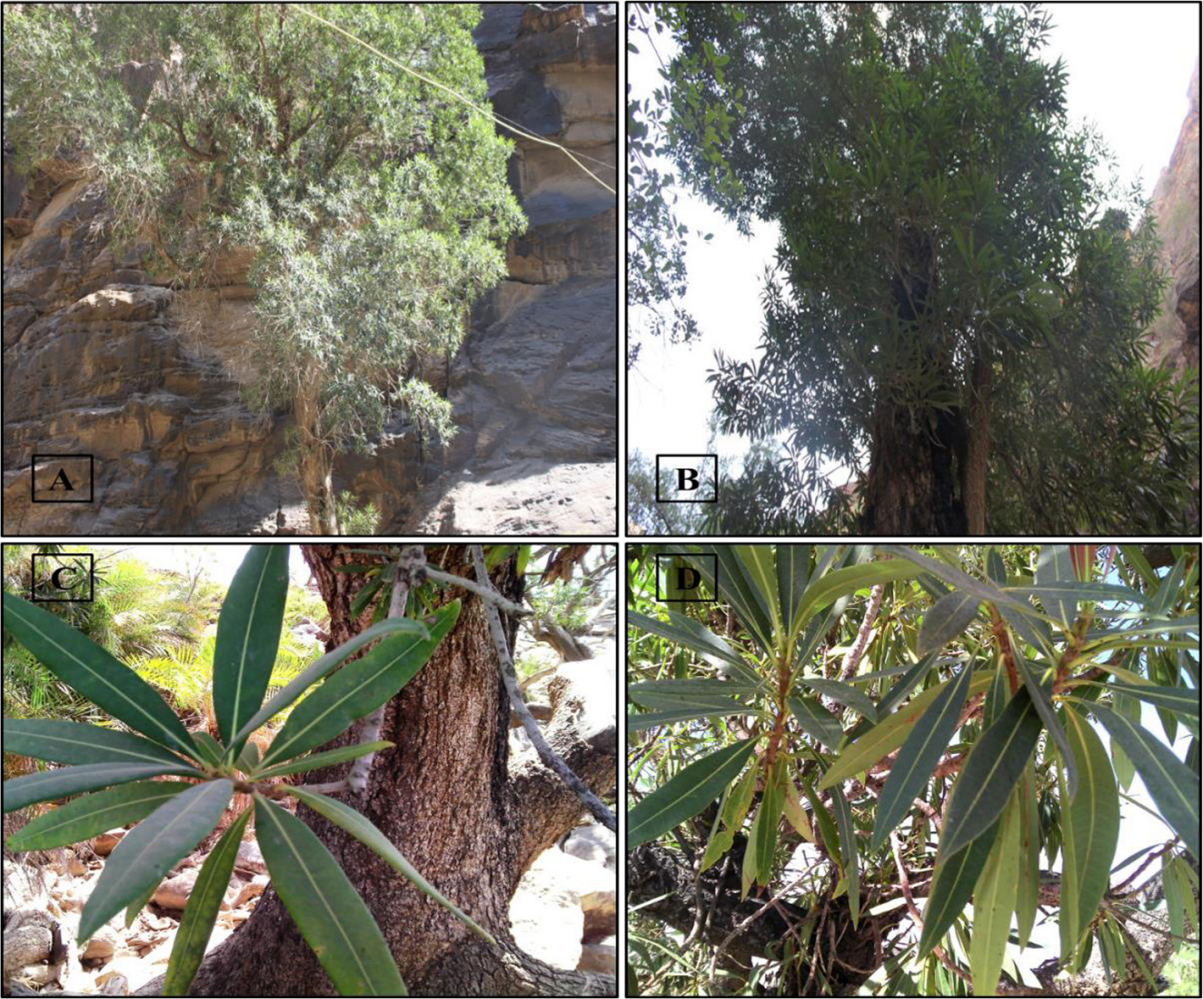
***Breonadia salicina*** **. (A–B)** Trees from Wadi Lejib, Jizan Province **(A)** and Rabuaa, Asir Province **(B)**. **(C–D)** Leaf morphology.

*Breonadia* Ridsdale (Rubiaceae) is a monotypic genus distributed in tropical Africa and the southern Arabian Peninsula. *Breonadia salicina* (Vahl) Hepper & JRI Wood (Figure 1) is considered to be one of the most critically endangered plant species of southwestern Saudi Arabia [2–4]. Reaching up to 40 m in height and 2 m in diameter, trees of *B. salicina* usually grow along high escarpments from 500 to 2000 m above sea level, near the banks, or in the water of permanent streams and rivers [3,5]. *B. salicina* is monoecious with small, pale mauve and sweetly scented flowers. They are present in compact round axillary heads up to 40 mm in diameter on long slender stalks up to 60 mm, with 2 leaf-like bracts along their length. All floral parts are in fives, expanding into a funnel-shaped crag and 5-lobed cup-shaped disc. The stamens are inserted in the crag of the tube projecting from the spout. The ovary grows in the leaf from November to March with two chambers and sprightly yellow balls [6]. *Breonadia salicina* is a potentially medicinally and economically valuable plant that has been used for the treatment of many human diseases. Because of the presence of numerous secondary metabolites, the entire plant is widely used to treat arthritis, diabetes, diarrhea, cancer, headaches, gastrointestinal illness, fevers, wounds, ulcers, and bacterial and fungal infections [7–9]. In South Africa, *B. salicina* bark is used for stomach complaints and as an astringent [10], and root decoctions are used for the treatment of tachycardia [11]. The wood is yellowish, hard, heavy, very durable, termite resistant, and oily to the touch [6], making it an excellent, highly valued timber for furniture and house construction. Furthermore, *B. salicina* is a well-known firewood in Tanzania [12].

Assessment of within- and between-population genetic diversity of rare and endangered plants not only enhances our understanding of population dynamics, adaptation, and evolution, but also provides useful information for the development of conservation strategies [13]. Such an assessment is important because a species’ long-term survival and evolution depends on the maintenance of sufficient genetic variability within and among populations to accommodate new selection pressures brought about by environmental changes [14]. Molecular analysis of genetic variation among individuals of a population can be used to monitor the genetic variability of a declining population and to assess genetic fragmentation outcomes of surviving populations [15,16].

In recent decades, a series of techniques involving genetic markers have been developed to analyze and estimate genetic diversity. Compared with other genetic markers, DNA-based markers have superior reproducibility for the evaluation of genetic diversity. Among various marker systems, the inter-simple sequence repeat (ISSR) marker system uses repeat-anchored primers to amplify DNA sequences between two inverted simple sequence repeats [17]. ISSR markers are reproducible under a variety of conditions at relatively low expense, and enable the use of higher primer annealing temperatures and the generation of longer sequences than other markers. Because ISSRs are dominant markers, allelic variation cannot be detected, as homozygous and heterozygous states cannot be distinguished at individual loci [18]. Nevertheless, these markers have been successfully used in hundreds of studies for the assessment of genetic diversity of endangered plant species [19–21].

Comparisons with common congeners may decrease the contradictory effects of phylogeny [22,23]. To determine whether a rare species indeed exhibits low genetic diversity, it is therefore advantageous to compare genetic diversity measures in the rare species with those of a more widespread, common congener [24]. As mentioned above, however, *Breonadia* is a monotypic genus; to solve this problem, comparison with widespread *B. salicina* in Yemen can be used as an alternative for better understanding of genetic diversity.

Literature on *B. salicina* is limited, and information regarding its genetic basis is lacking. As a consequence, the purpose of this study was to investigate patterns of genetic diversity within and among natural populations of *B. salicina* in Saudi Arabia using ISSR markers and to compare their levels of genetic diversity with those of a population from Yemen, where the species is more widespread. Informed by the results, we also aimed to address possible strategies for long-term conservation of *B. salicina*.

## Methods

### Endangered plant survey

Previous information regarding *B. salicina* population levels and localities was insufficient. Therefore, to understand the spatial distribution and current state of this endangered plant species, we initially conducted searches at sites where *B. salicina* had been most recently collected and for which usable locality information was available. Information was obtained from King Saud University Herbarium records, site data from vegetation mapping, and botanists. A qualified botanist subsequently conducted a thorough survey of similar habitats in other areas for *B. salicina*. In the case of *B. salicina*, the most suitable habitat—along 500- to 2000-m high escarpments and above permanent streams and rivers—is characteristic of many sites in southwestern Saudi Arabia.

### Collection of plant material

**Figure 2:**
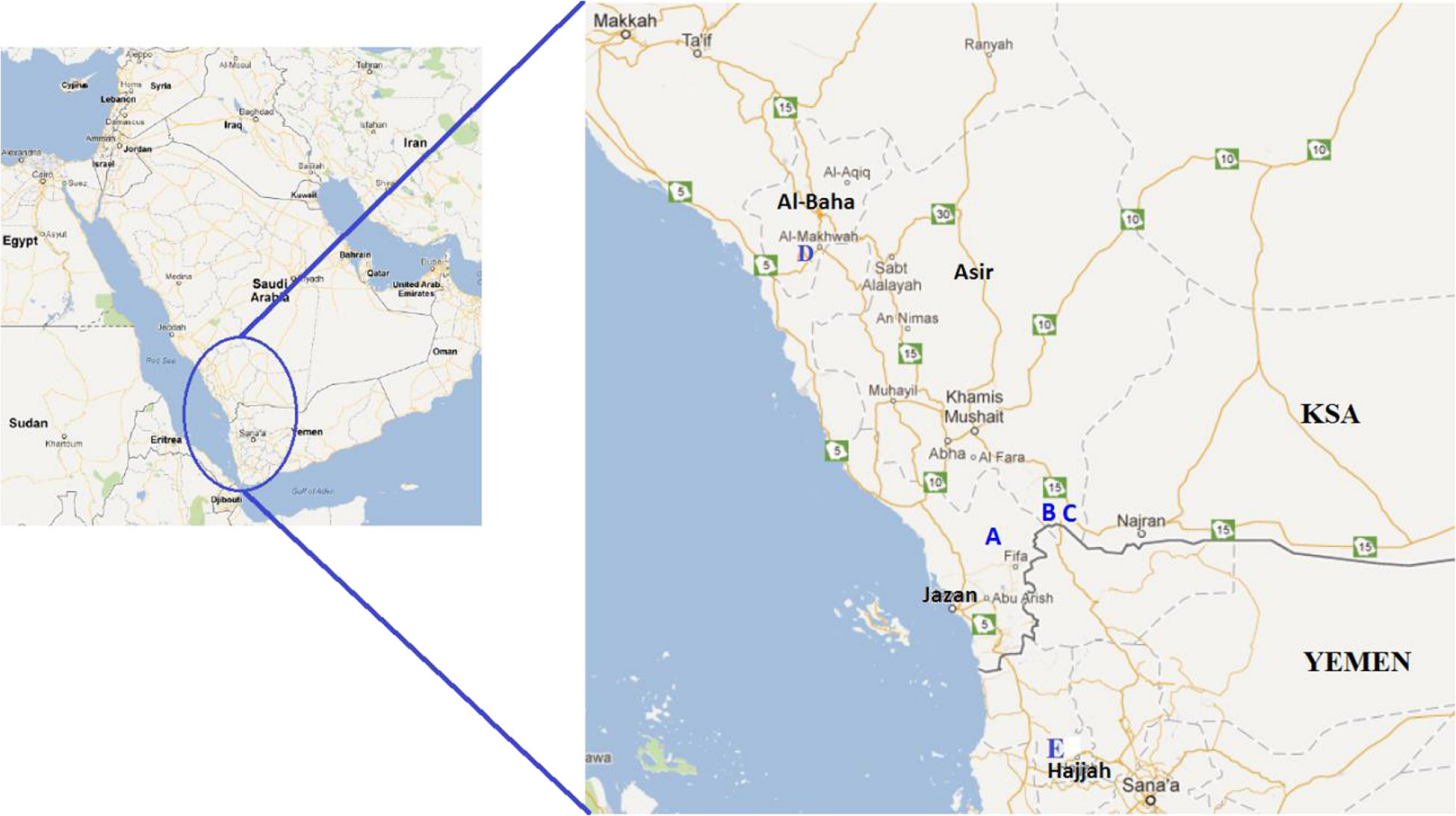
**Map showing locations of the populations of** ***Breonadia salicina*** **in Saudi Arabia and Yemen sampled in this study. (A)** Population 1: Wadi Lejib; **(B)** Population 2: Rabuaa, “Wadi Afkah”; **(C)** Population 3: Rabuaa, “Main Entrance”; **(D)** Al-Baha single individual; **(E)** Yemeni population.

Following the survey, leaf samples of fully-grown trees of *B. salicina* were collected from most individuals present in three fragmented natural populations located in southwestern Saudi Arabia and a single individual plant found in Al-Baha, Saudi Arabia. The remaining individuals (nine individuals) in the same populations were not sampled because of the difficulty of reaching their positions. For better comparison of genetic diversity, we also sampled a population from Yemen, as the tree is widely distributed in that country (Figure 2). After collection and labeling, all samples were preserved in silica gel until DNA isolation. Identification based on leaf and fruit morphological characters was performed at the Department of Botany and Microbiology, College of Science, King Saud University, Riyadh, and vouchers were deposited in the herbarium (Table 1).

**Table 1:** **Sampled populations of** ***Breonadia salicina*** **and their locations**

**Populations**	**Location**	**Province (region)**	**Number of sampled individuals**	**Number of individuals within population**	**Coordinates**	**Elevation (m)**
**Population 1**	Wadi Lejib	Jizan (KSA)	6	9	17° 36′ N	1610
					42° 56′ E	
**Population 2**	Rabuaa (Wadi Afkah)	Asir (KSA)	7	11	17° 33′ N	1680
					43° 18′ E	
**Population 3**	Rabuaa (Main Entrance)	Asir (KSA)	5	7	17° 34′ N	1715
					43° 19′ E	
**Al-Baha Individual**	Jabal Shada Al-Aala	Asir (KSA)	1	1	19° 51′ N	1060
					41° 18′ E	
**Yemeni population**	Mapyan	Hajjah (Yemen)	8	15	15° 43′ N	1810
					43° 34′ E	

### Extraction and purification of genomic DNA

Genomic DNA was isolated and purified using a modification of the cetyltrimethylammonium bromide CTAB method [25].

### PCR amplifications and ISSR profiling

ISSR primers were synthesized by Macrogen (Seoul, South Korea) and Sigma (UK). A set of 43 ISSR primers were screened for reproducibility and suitability for genetic diversity analysis after optimization of primer annealing temperatures according to their Tm values. DNA was diluted to a working concentration of 20 ng/μl. Amplifications were performed in 25-μl volumes using PCR beads (GE Healthcare, Spain) [26] on a Veriti 96-well thermocycler (Applied Biosystems), with the tubes vortexed and briefly spun (≈10 s) after addition of all components. PCR products were separated on a 1.3% agarose gel in 1× Tris/Borate/EDTA buffer TBE containing 0.5 μg/ml ethidium bromide and electrophoresed at 5 V/cm. Gels were photographed using a Syngene bio-imaging gel documentation system (Ingenius).

### Analysis of ISSR banding patterns

For determination of genetic diversity, banding patterns generated by the ISSR primers were analyzed by comparing bands present in various lanes. We scored the most prominent and distinct bands, assigning for each band a ‘1’ if present and ‘0’ if absent. To assess genetic diversity, basic parameters including Nei’s gene diversity index (*h*), the Shannon index (*I*), percentage of polymorphic loci (PPL), population diversity (*H*_S_), total gene diversity (*H*_T_) within populations, and inter-population differentiation (*G*_ST_) [27] were calculated from the data using the program POPGENE 1.32 [28]. A non-parametric analysis of molecular variance (AMOVA) was used to investigate the partitioning of genetic variation within and among regional populations using GenAlEx 6.5 software [29,30]. The generated data were analyzed with the NTSYS-pc 2.21 software package [31] to evaluate genetic similarity and phylogenetic relationships within and among populations using the Unweighted Pair Group Method with Arithmetic Mean (UPGMA) algorithm [32].

## Results and discussion

### Current status of *B. salicina* in Saudi Arabia

Our survey revealed that only one wild population remained in Wadi Lejib (Jizan Province) from previously reported locations, and that one had vanished in Wadi Fadah (Jizan Province). Similarly, our recent exploration discovered only one surviving individual of the formerly [33] thin (three- to four-tree) population of *B. salicina* in Al-Baha Province. On the other hand, two wild populations were newly identified and sampled from Asir Province in southwestern Saudi Arabia. The few known relict populations in the southwestern part of the country contained less than 30 individuals; no young seedlings were observed in these populations, except in Wadi Lejib (Jizan Province) (Table 1). Although some recreational activities take place in Wadi Lejib, many endangered plants—including this species—are thriving there [3]. Threats to *B. salicina* are due to several factors: environmental pressures, anthropogenic activities, including the current low rate of natural management, and increased habitat fragmentation and deterioration. Regardless of the reasons, an important consequence is that reductions in population size are leading to a situation of inbreeding, reducing within-population genetic diversity.

### Genetic diversity

**Figure 3:**
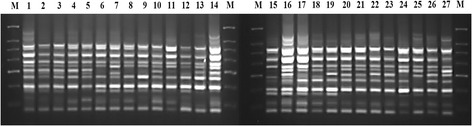
**ISSR profile resulting from amplification of genomic DNA of Saudi Arabian and Yemeni** ***Breonadia salicina*** **accessions with primer UBC-10.** M: 100-bp ladder; 1–27, accessions of *B. salicina.*

The application of ISSR markers in the present study revealed genetic diversity levels that were low within populations but relatively high among populations. Out of the 43 ISSR primers tested, 14 (Table 2) produced well-reproducible, scorable patterns. The 14 primers generated a total of 211 prominent, reproducible DNA bands ranging in length from 185 to 2,200 bp (Figure 3); 68 of these were polymorphic across all accessions, reflecting poor allelic diversity in the sampled populations. Bands per primer ranged from 10 (UBC 42) to 25 (UBC 34). The average number of loci per primer was 15.1, while the average number of polymorphic loci per primer was 4.9. The largest number of polymorphic loci was 13 (52%), generated from primer UBC 34, and the lowest, from primer UBC 26, was 1 (9.1%). At the population level, a low range of genetic diversity was observed. PPL per population ranged from 17.1 to 23.7%, *h* varied from 0.0714 to 0.0968, and *I* ranged from 0.1031 to 0.1405 (Table 3).

**Table 2:** **ISSR primers used in this study**

**S.N**	**Name**	**Sequence**
**1**	UBC-10	GAG AGA GAG AGA GAG AT
**2**	UBC-11	GAG AGA GAG AGA GAG AC
**3**	UBC-12	GAG AGA GAG AGA GAG AA
**4**	UBC-18	CAC ACA CAC ACA CAC AG
**5**	UBC-25	ACA CAC ACA CAC ACA CT
**6**	UBC-26	ACA CAC ACA CAC ACA CC
**7**	UBC-27	ACA CAC ACA CAC ACA CG
**8**	UBC-34	AGA GAG AGA GAG AGA GYT
**9**	UBC-35	AGA GAG AGA GAG AGA GYC
**10**	UBC-41	GAG AGA GAG AGA GAG AYC
**11**	UBC-42	GAG AGA GAG AGA GAG AYG
**12**	UBC-48	ACA CAC ACA CAC ACA CYT
**13**	UBC-49	ACA CAC ACA CAC ACA CYA
**14**	UBC-50	ACA CAC ACA CAC ACA CYG

**Table 3:** **Genetic diversity within Saudi Arabian and Yemeni natural populations of** ***Breonadia salicina*** **based on ISSR markers**

**Population**	**# of Polymorphic Loci**	**PPL**	***h***	***I***
**Pop. 1**	50	23.7	0.097 (0.1817)	0.141 (0.2597)
**Pop. 2**	49	23.2	0.090 (0.1741)	0.132 (0.2502)
**Pop. 3**	36	17.1	0.071 (0.1629)	0.103 (0.2325)
**Average**	45	21.3	0.086	0.125
Pop. Yemen	51	24.2	0.094 (0.179)	0.137 (0.255)

Standard deviations are in parentheses. *h*, Nei’s gene diversity; *I*, Shannon’s information index; PPL, Percentage of polymorphic loci.

At the species level, PPL, *h*, and *I* were 32.2%, 0.116, and 0.172, respectively (Table 4). This level of genetic diversity is low compared with that uncovered in many other ISSR-based studies of endangered and threatened plants [34–36]. The average PPL in *B. salicina* across the three natural Saudi Arabian populations and the Yemeni population at the species level was considerably lower than that reported in *Psychotria ipecacuanha*, *Galium cracoviense*, and *Palicourea coriacea*, other species in the Rubiaceae [37–39]. Mean *h* within the three sampled Saudi Arabian populations of *B. salicina* showed considerably lower diversity (*h* = 0.086) than that obtained in many common species using RAPD markers (*h* = 0.22 or 0.23) [40]. The low genetic diversity of *B. salicina* may be due to its breeding system, restricted geographic attribution, gene flow, genetic drift, and/or natural selection. The type of breeding system is the main factor affecting genetic diversity both among and within populations [41,42]. At present, no comprehensive studies of the *B. salicina* mating system have been conducted. The genetic diversity found in *B. salicina* at the population level (*h* = 0.0861) is lower than the average for both outcrossing-plant species and mixed breeding (*h* = 0.27 and 0.18, respectively) and closely similar to that of self-pollinating plants (*h* = 0.091) using the same data source [40]. Based on this result, the genetic diversity of *B. salicina* is more compatible with selfing behavior, which often reduces within-population genetic variation and increases it among populations. In addition, habitat fragmentation leads to decreased genetic diversity and increased inter-population divergence, likely the result of restricted gene flow among fragmented populations, increased inbreeding, and random genetic drift within populations [43]. Small-sized populations have less genetic diversity than large-sized ones, as pollinators are more attracted to the latter [44–46].

**Table 4:** **Genetic diversity among Saudi Arabian populations of** ***Breonadia salicina*** **estimated using ISSR markers**

	***h***	***I***	**H**_**T**_	**H**_**S**_	**G**_**ST**_	**Number of polymorphic loci**	**PPL**
**ISSR**	0.116 (0.183)	0.172 (0.266)	0.116 (0.034)	0.086 (0.021)	0.256	68	32.2

The individual sampled from Al-Baha is excluded. Standard deviations are in parentheses. *h*, Nei’s gene diversity; *I*, Shannon’s Information index; *H*_T_, total gene diversity; *H*_S_, population diversity; *G*_ST_, Coefficient of genetic differentiation; PPL, Percentage of polymorphic loci.

On the other hand, ISSR primers generated a total of 51 polymorphic bands, with an average of 3.6, from the Yemeni population. The largest number of polymorphic bands, 9, was generated twice—from primers UBC 34 (36%) and 50 (56.3%); the lowest number, 1 (8.3%), was obtained using primer UBC 25. Upon combination of ISSR data from Yemeni and Saudi Arabian populations, a high level of genetic diversity was expected within the Yemeni population. The opposite was true, however, with low genetic diversity observed within the Yemeni population with respect to PPL, *h*, and other evaluated parameters, similar to the Saudi Arabian populations. PPL, *h*, and *I* in the Yemeni population were 24.2%, 0.094, and 0.137, respectively (Table 3).

### Genetic structure

In population genetics, gene differentiation based on *G*_ST_ values is classified as low (<0.05), medium (0.05–0.15), or high (>0.15) [47]. The *G*_ST_ value uncovered for *B. salicina* (0.256) would thus be considered high; by indirect comparison, however, it was much lower than values previously reported using ISSR markers (0.34) [40].

These findings raise the possibility that all present-day *B. salicina* populations were formerly part of a single population that became fragmented owing to anthropogenic activities and various ecological factors. An alternative, more likely hypothesis is that these populations were larger in the past and were probably bordered by intervening populations (which are now extinct, e.g., Wadi Fadah) that ensured low levels of gene diversity coupled with this level of genetic differentiation.

The calculated *G*_ST_ value for *B. salicina* was supported by an AMOVA, which indicated that 17% of the total variation was partitioned between populations among regions. When populations were analyzed without grouping, AMOVA indicated that 84.86% of the total variation in the studied populations was due to differences within populations, with the remaining 15.14% accounted for by among-population differences (Table 5).

**Table 5:** **Distribution of genetic variability within and between Saudi Arabian** ***Breonadia salicina*** **populations and regions measured by AMOVA of ISSR data**

	**Source of variance**	**Df**	**Sum of square**	**Variance component**	**% total of variance**	**Significance**
**1. Groups based on geographical origin**	Variance among regions	1	19.972	0.000	0%	
	Variance among populations	1	23.617	2.236	17%	P < 0.001
	Variance within populations	15	158.633	10.576	83%	
	Total	17	202.222	12.811	100%	
**2. Without grouping**	Variance among populations	2	43.589	1.887	15.14%	P < 0.001
	Variance within populations	15	158.633	10.576	84.86%	
	Total	17	202.222	12.463	100%	

df, degrees of freedom.

When groups were based on geographic origin (i.e., provinces of sampled populations; Table 1), among-region, among-population, and within-population contributions to the observed variance were 0%, 17%, and 83%, respectively. The null value of the variance among regions might be due to the proximity of different populations.

### Genetic relationships among *B. salicina* populations

Based on ISSR allele frequencies, Nei’s genetic identity (IN) values of the Saudi Arabian populations had a very narrow range, varying from 0.9514 to 0.9705, and pairwise genetic distances were between 0.0299 and 0.0498 (Table 6). Genetic identity based on IN was highest between populations 1 and 2 and lowest between populations 1 and 3, although these values were nearly identical.

**Table 6:** **Nei’s unbiased measures of genetic identity and genetic distance between Saudi Arabian and Yemeni** ***Breonadia salicina*** populations based on ISSR data*

**Pop ID**	**1**	**2**	**3**	**Yemen**
**1**	****	0.9705	0.9514	0.9210
**2**	0.0299	****	0.9572	0.9354
**3**	0.0498	0.0437	****	0.9434
**Yemen**	0.0823	0.0667	0.0583	****

* Nei’s genetic identity and genetic distance is indicated above and below diagonals, respectively.

**Figure 4:**
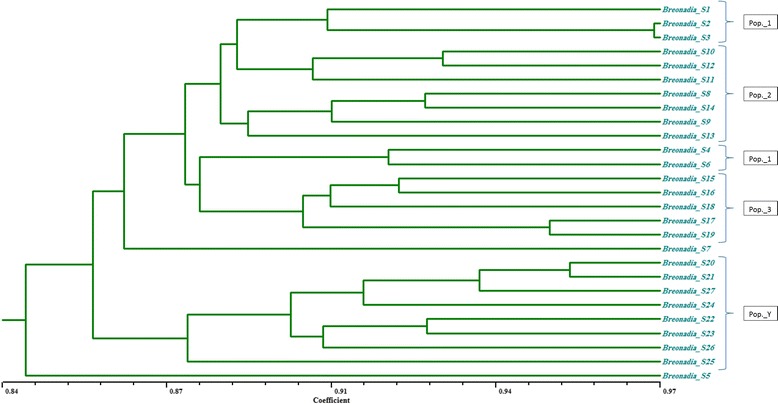
**Dendrogram obtained from UPGMA cluster analysis of 27 accessions of** ***Breonadia salicina*** **.** The scale bar corresponds to genetic distance based on Jaccard’s similarity coefficient.

These close genetic identities suggest that all three Saudi Arabian populations had a recent common ancestor, an inference further supported by the results of the UPGMA analysis (Figure 4). Moreover, the three *B. salicina* populations share similar habitats; such a situation tends to result in similar population characteristics, as a clear association exists between a population’s characteristics and its environment [48,49]. The similar habitats of these populations sharing very close genetic identities also support the hypothesis that these populations have only recently become separated.

We evaluated the genetic relationships of the 19 *B. salicina* accessions from Saudi Arabia along with the eight Yemeni accessions based on Jaccard’s similarity coefficient using UPGMA cluster analysis [32] (Figure 4). The 19 Saudi Arabian *B. salicina* accessions were divided into two major clusters. Cluster I comprised 10 accessions from populations 1 and 2, and cluster II contained seven accessions from population 3 and 2 outliers from population 1 (*Breonadia*_S4 and *Breonadia*_S6). In addition, a sample from Al-Baha (*Breonadia*_S7), the geographically most distant locality, was also the most genetically distant accession, forming its own clade. One accession (*Breonadia*_S5) from population 1 was widely separated from the rest of the Saudi Arabian accessions, suggesting that population 1 was the ancestral population of *B. salicina*. Although genetic similarity based on Jaccard’s similarity coefficients was highest between populations 1 and 2 and lowest between populations 1 and 3, genetic similarities among all three populations were nearly identical. Jaccard’s similarity coefficients ranged from 82.76 to 94.82% among the *B. salicina* accessions, indicating a narrow genetic diversity (Table 7). An accession of population 3 (*Breonadia*_S15) had the lowest genetic similarity (82.76%) to the other studied accessions. The cause of this higher genetic diversity is unknown, but is presumably due to environmental factors prevailing at the higher altitude (1,715 m) of population 3.

**Table 7:** **Similarity matrix generated using NTSYS-pc software based on ISSR data from 27 accessions of** ***Breonadia salicina***

	***Breo nadia S1***	***Breo nadia S2***	***Breo nadia S3***	***Breo nadia S4***	***Breo nadia S5***	***Breo nadia S6***	***Breo nadia S7***	***Breo nadia S8***	***Breo nadia S9***	***Breo nadia S10***	***Breo nadia S11***	***Breo nadia S12***	***Breo nadia S13***	***Breo nadia S14***	***Breo nadia S15***	***Breo nadia S16***	***Breo nadia S17***	***Breo nadia S18***	***Breo nadia S19***	***Breo nadia S20***	***Breo nadia S21***	***Breo nadia S22***	***Breo nadia S23***	***Breo nadia S24***	***Breo nadia S25***	***Breo nadia S26***	***Breo nadia S27***
S1	1.000																										
S2	0.899	1.000																									
S3	0.910	*0.969*	1.000																								
S4	0.884	0.874	0.875	1.000																							
S5	0.847	0.864	0.866	0.849	1.000																						
S6	0.893	0.873	0.884	0.916	0.848	1.000																					
S7	0.859	0.849	0.850	0.861	0.843	0.851	1.000																				
S8	0.894	0.893	0.894	0.869	0.850	0.897	0.871	1.000																			
S9	0.898	0.878	0.879	0.844	0.863	0.891	0.866	0.902	1.000																		
S10	0.889	0.898	0.899	0.864	0.855	0.892	0.858	0.913	0.878	1.000																	
S11	0.878	0.878	0.879	0.872	0.853	0.871	0.874	0.892	0.857	0.907	1.000																
S12	0.888	0.888	0.879	0.835	0.863	0.891	0.847	0.902	0.916	0.927	0.896	1.000															
S13	0.866	0.884	0.866	0.859	0.869	0.849	0.862	0.869	0.892	0.865	0.854	0.873	1.000														
S14	0.910	0.871	0.873	0.856	0.866	0.874	0.877	0.923	0.908	0.899	0.860	0.879	0.904	1.000													
S15	0.899	0.879	0.881	0.846	0.828	0.873	0.858	0.893	0.859	0.889	0.887	0.888	0.865	0.862	1.000												
S16	0.881	0.871	0.881	0.865	0.856	0.903	0.868	0.884	0.860	0.909	0.869	0.869	0.856	0.900	0.918	1.000											
S17	0.885	0.853	0.867	0.879	0.833	0.888	0.891	0.870	0.855	0.866	0.873	0.846	0.889	0.876	0.894	0.904	1.000										
S18	0.886	0.876	0.877	0.852	0.861	0.870	0.882	0.881	0.866	0.867	0.884	0.847	0.881	0.877	0.914	0.896	0.910	1.000									
S19	0.904	0.866	0.886	0.898	0.833	0.917	0.863	0.908	0.864	0.904	0.892	0.874	0.879	0.895	0.894	0.904	0.948	0.891	1.000								
S20	0.875	0.874	0.886	0.878	0.850	0.868	0.871	0.888	0.864	0.874	0.882	0.873	0.879	0.876	0.865	0.884	0.889	0.890	0.889	1.000							
S21	0.869	0.859	0.851	0.872	0.834	0.871	0.865	0.872	0.848	0.859	0.876	0.857	0.872	0.869	0.878	0.907	0.892	0.865	0.883	0.952	1.000						
S22	0.853	0.824	0.835	0.837	0.809	0.836	0.840	0.876	0.841	0.843	0.850	0.832	0.856	0.872	0.843	0.872	0.886	0.849	0.886	0.876	0.919	1.000					
S23	0.863	0.834	0.836	0.838	0.819	0.837	0.850	0.876	0.851	0.853	0.860	0.851	0.867	0.873	0.843	0.854	0.858	0.850	0.877	0.896	0.910	0.924	1.000				
S24	0.829	0.838	0.839	0.832	0.832	0.840	0.835	0.870	0.836	0.838	0.845	0.827	0.851	0.848	0.847	0.867	0.871	0.845	0.881	0.890	0.914	0.897	0.878	1.000			
S25	0.878	0.822	0.824	0.843	0.816	0.824	0.837	0.882	0.857	0.840	0.838	0.829	0.844	0.879	0.840	0.841	0.864	0.847	0.873	0.872	0.876	0.869	0.870	0.884	1.000		
S26	0.858	0.811	0.822	0.851	0.814	0.832	0.845	0.862	0.809	0.838	0.855	0.809	0.843	0.859	0.838	0.848	0.872	0.845	0.881	0.871	0.894	0.918	0.888	0.872	0.874	1.000	
S27	0.884	0.855	0.866	0.859	0.840	0.858	0.880	0.888	0.863	0.874	0.881	0.854	0.869	0.894	0.864	0.884	0.879	0.861	0.888	0.927	0.941	0.925	0.915	0.930	0.891	0.910	1.000

**Figure 5:**
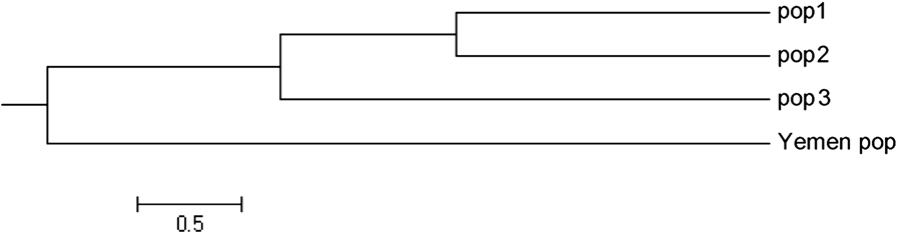
**UPGMA dendrogram showing relationships between Saudi Arabian and Yemeni populations of** ***Breonadia salicina*** **.**

On the other hand, based on genetic distance (Tables 6 and 7), the Yemeni population was more differentiated and formed a separate clade in the UPGMA dendrogram (Figures 4 and 5). The lower genetic diversity of the Yemeni population in spite of the species’ wider distribution in that country may be due to the presence of optimal growing conditions for *B. salicina* (i.e., high escarpments and abundant rainfall) without any external disturbance. In addition, the plant is cultivated in Yemen for wood and timber [50,51].

### Implications for *B. salicina* conservation

Drawing on our study results, we suggest a possible management strategy for the conservation of *B. salicina*. The first component of our proposed strategy concerns the mating system of *B. salicina*, suggested in this study to involve self-pollination. To verify this finding, further investigation is urgently needed. The outcome of such a study would have important implications for *B. salicina* conservation and management, as knowledge of the mating system is a major factor in the elucidation of genetic variability levels at both species and population levels [52].

A second important consideration for restoration of endangered and rare species is population size [53]. Establishment of on-site protection zones for *B. salicina* to reduce the impact of human activities would allow its habitats to increase in size through natural regeneration to reach effective population sizes. The construction of an *in situ* conservation area to preserve all extant populations would also maintain most existing genetic variation. Although losses of any one population or individual tree at specific locations may not cause an immediate decrease in genetic diversity, such losses have dangerous long-term consequences.

Third, plants can be introduced from other populations via appropriate propagation and seedling management to increase the chance of gene exchange and recombination and to improve the level of genetic diversity over time. In Tanzania, effective management procedures for *B. salicina* have included seed germination, which is more successful under moist conditions, and placing cuttings and wildlings in mud, the most frequently used method. Wildlings appear to have a higher survival rate than nursery seedlings, which need to be at least 1 m high to survive transplantation [12].

A fourth suggested management practice is establishment of an *ex situ* conservation program to capture most of the detected genetic variability and to increase genetic diversity by crossing different populations. Fifth, collection of seeds and germplasm in botanical gardens or other institutions may be of significant practical value for the conservation of genetic diversity. Sixth, establishment of a regeneration system for this endangered plant via tissue culture would not only guarantee its *ex situ* conservation and sustainable survival, but would also enhance its *in situ* conservation. A seventh component of the action plan is the education and involvement of local people. Finally, as genetic information alone will still not provide a sufficient solution, demographic and ecological perspectives must be taken into consideration before drawing up and implementing a conservation strategy.

## Conclusions

The molecular markers used in this study were found to be effective for the assessment of *B. salicina* genetic diversity. Observed low levels of within-population genetic diversity coupled with the relatively high genetic differentiation detected among Saudi Arabian *B. salicina* populations suggest the hypothesis that these populations were formerly part of a single population that has become fragmented as a result of biotic and abiotic factors in the natural habitat. Alternatively, current populations were larger in the past, with intervening, now-extinct populations (e.g., Wadi Fadah) ensuring lower levels of gene diversity. While both Saudi Arabian and Yemeni populations are marked by low genetic diversity, the presence of optimal growing conditions and cultivation in Yemen has contributed to the widespread distribution of *B. salicina* in that country. To broaden the genetic base of *B. salicina* and conserved this critically endangered plant species in Saudi Arabia, appropriate management and conservation strategies require implementation.
